# Patterns of human and porcine gammaherpesvirus-encoded BILF1 receptor endocytosis

**DOI:** 10.1186/s11658-023-00427-y

**Published:** 2023-02-21

**Authors:** Maša Mavri, Sanja Glišić, Milan Senćanski, Milka Vrecl, Mette M. Rosenkilde, Katja Spiess, Valentina Kubale

**Affiliations:** 1 Institute for preclinical sciences, Veterinary Faculty, Ljubljana, Slovenia; 2 Center for Multidisciplinary Research, Institute of Nuclear Sciences VINCA, University of Belgrade, Belgrade, Serbia; 3 Department of Biomedical Sciences, Faculty of Health and Medical Sciences, University of Copenhagen, Copenhagen, Denmark; 4 Present Address: Department of Virus and Microbiological Special Diagnostics, Statens Serum Institute, Copenhagen, Denmark

**Keywords:** vGPCR, BILF1, Endocytosis, Internalization, Dynamin, Caveolin, β-Arrestin, EBV, PLHV1-2

## Abstract

**Background:**

The viral G-protein-coupled receptor (vGPCR) BILF1 encoded by the Epstein–Barr virus (EBV) is an oncogene and immunoevasin and can downregulate MHC-I molecules at the surface of infected cells. MHC-I downregulation, which presumably occurs through co-internalization with EBV-BILF1, is preserved among BILF1 receptors, including the three BILF1 orthologs encoded by porcine lymphotropic herpesviruses (PLHV BILFs). This study aimed to understand the detailed mechanisms of BILF1 receptor constitutive internalization, to explore the translational potential of PLHV BILFs compared with EBV-BILF1.

**Methods:**

A novel real-time fluorescence resonance energy transfer (FRET)-based internalization assay combined with dominant-negative variants of dynamin-1 (Dyn K44A) and the chemical clathrin inhibitor Pitstop2 in HEK-293A cells was used to study the effect of specific endocytic proteins on BILF1 internalization. Bioluminescence resonance energy transfer (BRET)-saturation analysis was used to study BILF1 receptor interaction with β-arrestin2 and Rab7. In addition, a bioinformatics approach informational spectrum method (ISM) was used to investigate the interaction affinity of BILF1 receptors with β-arrestin2, AP-2, and caveolin-1.

**Results:**

We identified dynamin-dependent, clathrin-mediated constitutive endocytosis for all BILF1 receptors. The observed interaction affinity between BILF1 receptors and caveolin-1 and the decreased internalization in the presence of a dominant-negative variant of caveolin-1 (Cav S80E) indicated the involvement of caveolin-1 in BILF1 trafficking. Furthermore, after BILF1 internalization from the plasma membrane, both the recycling and degradation pathways are proposed for BILF1 receptors.

**Conclusions:**

The similarity in the internalization mechanisms observed for EBV-BILF1 and PLHV1-2 BILF1 provide a foundation for further studies exploring a possible translational potential for PLHVs, as proposed previously, and provides new information about receptor trafficking.

**Graphical Abstract:**

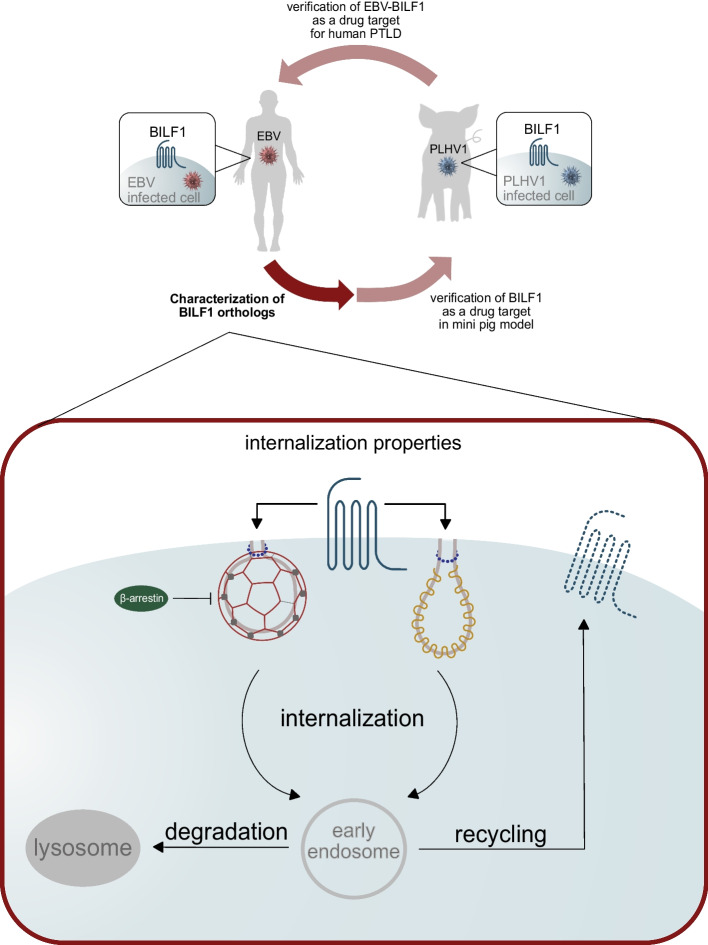

**Supplementary Information:**

The online version contains supplementary material available at 10.1186/s11658-023-00427-y.

## Background

Several herpesviruses encode G-protein-coupled receptors (vGPCRs), which are transmembrane proteins related to endogenous GPCRs, and have presumably been acquired from hosts over years of coevolution [1–3]. They functionally support viral replication and survival in the infected host by mediating immune evasion, promoting viral dissemination and cell transformation, or triggering pro-inflammatory responses [1–4].

The vGPCR BILF1 was first identified in human Epstein–Barr virus (EBV) (genera *Lymphocryptovirus*) [5–7] and later in nonhuman primate herpesviruses (genera *Lymphocryptovirus*) [8] and ungulate herpesviruses (genera *Macavirus*) (e.g., porcine lymphotropic herpesvirus 1, 2, and 3) [9, 10]. EBV-BILF1 has been extensively studied [5, 6], showing its transforming potential both in vitro and in vivo through Gα_i_-dependent signaling [11] and immunoevasive potential through the downregulation of major histocompatibility complex I (MHC-I) molecules from the cell surface of BILF1 transfected cells [12–14]. Its immunomodulatory function has been linked to the receptor’s ability to constitutively internalize [8, 12]. The mechanism underlying this process has not yet been studied in detail, but the importance of receptor trafficking has been suggested in this regard [14]. We have recently shown that BILF1 orthologs encoded by porcine lymphotropic herpesviruses (PLHVs) exhibit conserved signaling, constitutive internalization, and immunomodulatory properties, indicating the applicability of a porcine model to study EBV-related diseases and BILF1 as a potential therapeutic target [15]. Here, we further studied the endocytic mechanisms used by BILF1 receptors to describe their trafficking from the plasma membrane to the cell interior.

Most GPCRs are expressed at the cell surface and are mainly activated by extracellular stimuli (ligands) and subsequently transported intracellularly through endocytosis resulting in the waning of receptor-mediated signaling [16]. However, some are transported into the cytoplasm without initial ligand activation and are therefore constitutively internalized. The internalization of GPCRs from the cell surface requires specific proteins that selectively direct the receptors into the cell interior. Clathrin-mediated endocytosis is the best-studied endocytic pathway characterized by a specific clathrin triskelion coat around a newly formed vesicle [17, 18]. Several adaptor proteins, including adaptor protein 2 (AP-2) [19], β-arrestins [20, 21], epsin, and Eps15 [22], select the specific cargo and initiate the clathrin recruitment, membrane invagination, and complete vesicle formation at the plasma membrane. Besides, protein caveolin, together with the cavin and syndapin proteins, form specific, flask-shaped membrane invaginations in membrane areas rich in cholesterol and sphingomyelin [23]. However, unlike clathrin-mediated endocytosis, interactions with caveolin do not necessarily lead to receptor endocytosis as caveolins also affect the exocytosis and intracellular trafficking of GPCRs [24]. Both clathrin- and caveolin-rich vesicles are released from the plasma membrane by the large GTPase dynamin [25, 26]. Clathrin-coated vesicles fuse with early endosomes in the cytoplasm, where the cargo is then either recycled back to the plasma membrane through recycling endosomes [27] or degraded in the lysosomes [28]. In this way, cells can regulate the protein and lipid composition at the plasma membrane and thus regulate its response to the extracellular environment [29]. Although the lack of caveolae-specific cargo makes the studies challenging [30], internalized caveolae have been shown to fuse with early endosomes, further maturating into late endosomes and multivesicular bodies and eventually fusing with lysosomes [31, 32]. Furthermore, the major component of the caveolae, caveolin-1 was reported to co-localize with Rab11, a marker for recycling endosomes, indicating that caveolae may undergo a recycling pathway [33].

The present study describes the endocytic pathways and intracellular trafficking properties of BILF1 receptors encoded by EBV, PLHV1, and PLHV2 in HEK-293A cells using a novel real-time FRET-based internalization assay. By disrupting the process of clathrin vesicle formation using chemical inhibitor Pitstop2 and by inhibiting the function of wild-type dynamin using the dominant-negative mutant (DNM) Dyn K44A, we identified dynamin-dependent, clathrin-mediated endocytosis for BILF1 receptors. Furthermore, internalization of BILF1 receptors in HEK-293A β-arrestin 1/2 knockout cells (β-arr 1/2 KO) and additional BRET2 experiments and a bioinformatics approach (informational spectrum method—ISM) showed that β-arrestin recruitment is not essential for BILF1-mediated internalization. Additionally, inhibition of wild-type caveolin-1 by DNM Cav S80E also affected BILF1-mediated internalization, indicating the involvement of caveolin/raft-dependent endocytosis. Finally, in addition to the proposed recycling pathway, the BRET1 saturation assay indicated interactions between BILF1 receptors and Rab7, proposing that at least some BILF1 receptors are processed through the degradation pathway.

## Methods

### Constructs and cloning

For FRET-based real-time internalization, EBV-BILF1, PLHV1-BILF1, PLHV2-BILF1, and glucose-dependent insulinotropic polypeptide receptor (GIP-R) open reading frames were cloned into pcDNA5/FRT/TO-FLAG-SNAP vectors containing SNAP and FLAG tags at the N-terminus. For the BRET assay, BILF1 receptor constructs N-terminally tagged with a FLAG tag and C-terminally tagged with *Renilla* luciferase 8 (RLuc8) were purchased from GenScript (GenScript, Piscataway, NJ). Constructs of DNM caveolin-1 (Cav S80E) and dynamin-1 (Dyn K44A) were kindly provided by Prof. J.E. Pessin (Department of Physiology and Biophysics, University of Iowa, IA, USA) and Prof. M.G. Caron (Duke University Medical Center, NC, USA), respectively, and were described previously [34]. The human β-arrestin 2 N-terminally tagged with GFP^2^ (GFP^2^-βarr2) was purchased at PerkinElmer BioSignal, Inc. (Montreal, Ontario, Canada). The membrane-inserted GFP^2^-tagged construct (GFP^2^-17aa) was kindly provided by Dr. Rasmus Jørgensen (7TM Pharma A/S, Hørsholm, Denmark) and was described previously [35, 36]. EGFP-Rab7 was purchased from Addgene (Watertown, MA, USA).

### Cell culture and transfection

HEK-293 cells were purchased from European Collection of Cell Cultures (cat. no. 85120602). HEK-293A cells and CRISPR/Cas9-modified β-arrestin 1/2 knockout (β-arr KO) HEK-293A cells were kindly provided by Asuka Inoue (Tohoku University, Japan). All the cells were cultured at 37 °C and 10% CO_2_ in Dulbecco’s modified Eagle medium (DMEM; Invitrogen) containing 10% fetal bovine serum (FBS) and 1% penicillin–streptomycin. Cells were transfected using Lipofectamine 2000 (Invitrogen).

### FRET-based real-time internalization assay

HEK-293A cells (parental) or HEK-293A β-arrestin 1/2 knockout cells (β-arr KO) were transiently transfected with Lipofectamine 2000 to express SNAP-tagged EBV-BILF1, PLHV1-BILF1, PLHV2-BILF1, or SNAP-FRT (empty vector). Additionally, parental and β-arr KO HEK-293A cells transiently transfected with SNAP-tagged GIP-R were used as a positive control for agonist-induced (100 nm GIP) β-arrestin-dependent internalization. To determine specific internalization pathways, the DNM of dynamin-1 (Dyn K44A) or caveolin-1 (Cav S80E) was co-transfected in parental cells at various concentrations.

Cells, mixed with the transfection mixture, were seeded in poly-d-lysine (Sigma-Aldrich) precoated 384-well plates at a density of 16,000 cells per well. After 24 h, transfection medium was replaced with fresh growth medium. The next day, cells were incubated for 1 h with 100 nmol/L of cell-impermeable Tag-Lite SNAP-Lumi4Tb [donor; Cisbio, Codolet, France (SSNPTBX)] in Opti-MEM at 4 °C to prevent internalization during labeling. Afterwards, cells were washed four times using HBSS supplemented with 1 mmol/L CaCl_2_, 1 mmol/L MgCl_2_, and 20 mmol/L HEPES, pH 7.4. Then, 50 µmol/L of prewarmed (37 °C) fluorescein‐O′‐acetic acid [acceptor; Sigma‐Aldrich, Broendby, Denmark (88,596‐5MG‐F)] was added to the cells, and the measurements were recorded immediately after. Internalization was measured every 4 min for a total of 88 min at 37 °C in PerkinElmer EnVision 2104 Multilabel Reader using a 340 nm excitation filter. Emissions were detected using 520 nm (acceptor) and 615 nm (donor) emission filters. Results are presented as a ratio of donor over acceptor emissions (615/520 nm). The first value (timepoint 0) for each curve was used as a baseline. Control, empty-vector-transfected cells are presented on graphs and were used for normalization. Experiments were performed at least three times in triplicate. To compare the amount of internalization in the different conditions, the area under the curve (AUC) parameter was calculated as described previously [37] and was normalized to each BILF1 receptor AUC in the absence of inhibitors or DNMs. Receptor expression is represented by the donor values of the first measurement (timepoint 0 min) and is shown on graphs relative to each BILF1 receptor.

### *Bioluminescence resonance energy transfer (BRET) method*

BRET2 and BRET1 experiments were performed as previously described [35, 38, 39]. Briefly, HEK-293 cells were transiently co-transfected with constant amounts of the RLuc8-tagged BILF1 receptor constructs and increasing amounts of the GFP^2^-tagged β-arrestin 2, 17aa, or EGFP-tagged Rab7 using Lipofectamine LTX reagent. Forty-eight hours after transfection, 180 µL of resuspended cells at a density of ∼ 1.1 million cells/mL were distributed in 96-well microplates (white Optiplate; Packard BioScience, Meriden, CT, USA). Then, 10 µL of 100 µM coelenterazine 400A (BRET2) (Biotium, Fremont, CA, USA) or coelenterazine h (Thermo Fisher Scientific, Waltham, MA, USA) was added to each well using an injector. Sequential measurements of the emissions to measure the RLuc8 luminescence signal at 410 nm (BRET2) or 480 nm (BRET1) and the emissions of the light from excited GFP^2^ at 515 nm (BRET2) or EGFP at 540 nm (BRET1) were performed using a TriStar LB 942 microplate reader (Berthold Technologies, Bad Wildbad, Germany). Results are presented as ratios (515/410; BRET2 signal or 540/480; BRET1 signal) and expressed in milliBRET units (mBU); BRET ratio × 1000. The expression levels of RLuc8- and GFP^2^- or EGFP-tagged constructs for each experiment were assessed on the basis of total luminescence and fluorescence (measured on black plates, using excitation filter at 380 nm and emission filter at 515 nm). Measurements were performed in triplicate.

### Informational spectrum method (ISM)

According to the ISM approach, sequences (protein or nucleotide) are converted into signals by assigning numerical values to each constituent (amino acid or nucleotide). These values correlate to the electron–ion interaction potential (EIIP), a parameter that determines the electronic properties of amino acids and nucleotides. Fourier transform is then used to decompose the resulting signal into periodic functions, resulting in an output consisting of a series of frequencies and their amplitudes. The obtained frequencies correspond to the distribution of structural motifs with defined physicochemical characteristics that are responsible for the biological function of the sequence. By comparing the biological or biochemical function of proteins, we can detect code–frequency pairs that are specific to their common biological properties [40, 41]. The approach is not sensitive to the placement of the motifs and hence does not require prior sequence alignment. The ISM approach comprises two steps. First, the amino acids in the primary protein structure are represented by numbers corresponding to the EIIP (Additional file 1a, b). Second, the obtained numerical sequence is transformed by Fourier transformation into an informational spectrum (IS) (Additional file 1c). Each peak in the IS represents particular information encoded in the protein’s primary structure. Proteins that interact with a common target have been shown to share common frequencies in their IS. The principle of the ISM has been comprehensively described and applied for structure–function analysis of different proteins and for prediction of new protein interactors and identification of protein domains responsible for long-range interactions [42–44]. Viral and cellular GPCRs share similar pathological signaling networks, and protein–protein interaction data are commonly used for inferring specific signaling pathways [45, 46]. Proteins involved in similar signaling networks have been reported to share common information, represented by the IS frequencies [47].

### Immunoprecipitation

The immunoprecipitation assay was performed in HEK-293 cells, which were transiently transfected with RLuc8-tagged BILF1 receptors. Then, 48 h after transfection, cells were washed with cold PBS and were scraped and lysed for 20 min at 4 °C in NP-40 lysis buffer. Lysates were transferred to a cold tube, and the debris was removed by centrifugation. To control the transfection efficiency, 25 µL of the lysate was stored. The remaining lysate was incubated with monoclonal anti-AP-2 antibody (4 µg/mL) (Sigma-Aldrich) at 4 °C for 1 h. Thereafter, 50 µL of protein G agarose beads (Roche, Mannheim) were added, and the tubes were incubated at 4 °C overnight. The next day, the samples were centrifuged, and the immunoprecipitated complexes were washed extensively in NP-40 lysis buffer. After the final wash, the pellet was suspended in 550 µL of lysis buffer, and 180 µL was plated in white 96-well microplates. Total luminescence signal was measured in the presence of 10 µL of 100 µM coelenterazine 400A (Biotium) per well using a TriStar LB 942 microplate reader (Berthold Technologies). Results are presented as raw data with subtracted background. Experiments were performed three times.

### Data analysis and statistics

Data were analyzed using GraphPad Prism (9.3.1) and reported as the mean ± standard error of the mean (SEM). Statistical analysis was performed with GraphPad Prism using one-way analysis of variance (ANOVA; indicated in the figure legends). The statistical test was chosen on the basis of the data distribution determined using the normality test with GraphPad Prism. A *P*-value < 0.05 was considered statistically significant.

## Results

### Constitutive internalization of BILF1 receptors

**Fig. 1:**
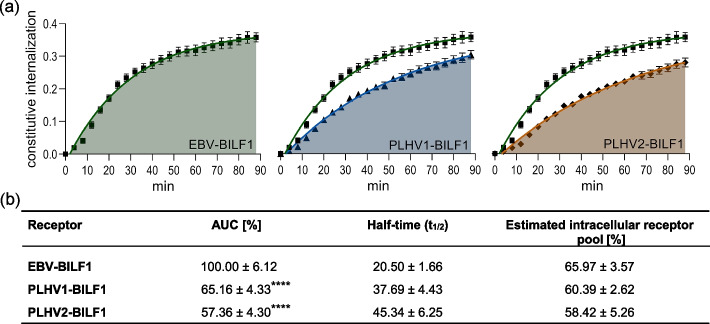
Constitutive internalization of BILF1 receptors. **a** SNAP-EBV-BILF1 (black square; green), SNAP-PLHV1-BILF1 (black circle; blue), and SNAP-PLHV2-BILF1 (black up-pointing triangle; orange) were expressed in HEK-293A cells. Internalization data are presented as nonlinear regression curves. **b** AUC values and half-time (*t*_1/2_) were determined using one-phase association analysis. The estimated intracellular receptor pool was calculated from receptor expression values (donor values). Data are shown as the mean ± SEM from 27 independent experiments carried out in triplicates. Statistical differences were determined using the Dunnett’s multiple comparisons one-way ANOVA test. ****P < 0.0001

The constitutive internalization of EBV-BILF1 and PLHV1-2-BILF1 receptors was first analyzed using a FRET-based RT internalization assay. The constitutive internalization properties were described and compared by calculating the area under the curve (AUC) parameter and their half-time values (*t*_1/2_). The AUC was normalized to EBV-BILF1 (100%), and the results showed ~ 35% and ~ 43% lower constitutive internalization for PLHV1-BILF1 and PLHV2-BILF1, respectively (Fig. 1a, b). These results are consistent with our previously reported data for EBV-BILF1 and PLHV1-2-BILF1 [15]. Furthermore, EBV-BILF1 also demonstrated the fastest internalization kinetics, with an estimated *t*_1/2_ of 20 min, whereas PLHV1 and PLHV2-BILF1 showed slower internalization with *t*_1/2_ values of ~ 37 and ~ 45 min, respectively (Fig. 1b). To estimate the number of receptors being recycled back to the plasma membrane, the difference in the amount of donor-labeled SNAP-tag receptors under the two labeling conditions was determined (Additional file 2). Labeling the receptors with the donor molecule at 4 °C prevented internalization and recycling during this step, which resulted in the labeling of surface-expressed receptors only (Additional file 2b, c). In contrast, labeling at 37 °C enabled normal receptor cycling during this step, which resulted in the labeling of all BILF1 receptors in the cell (designated as 100%) (Additional file 2b, c). Therefore, the differences in the donor signals correspond to the number of receptors located intracellularly and subsequently recycled or trafficked to the plasma membrane (Additional file 2b, c). This estimation was performed on the basis of previous reports of GPCR trafficking, using radioligand binding to follow ligand–receptor complexes [48, 49]. Comparing the donor values from both labeling conditions, we have observed a 65%, 60%, and 58% difference for EBV-BILF1, PLHV1, and PLHV2-BILF1 receptors, respectively, which represents the estimated intracellular pool of receptors.

### Internalization of BILF1 receptors is impaired by the DNM of dynamin-1 and the inhibitor Pitstop2

The importance of clathrin-mediated endocytosis for BILF1-mediated internalization, has not been previously investigated. To address this, a FRET-based real-time internalization assay was used with HEK-293A cells expressing SNAP-tagged EBV, PLHV1, and PLHV2 BILF1 receptors. The involvement of clathrin-mediated endocytosis was studied by disrupting the vesicle scission from the plasma membrane using the dominant-negative dynamin-1 mutant Dyn K44A (defective in GTP binding and hydrolysis), a previously described selective inhibitor of clathrin-mediated endocytosis [34, 50] and the chemical inhibitor Pitstop2, which stops the interaction between clathrin and amphiphysin [51].

**Fig. 2:**
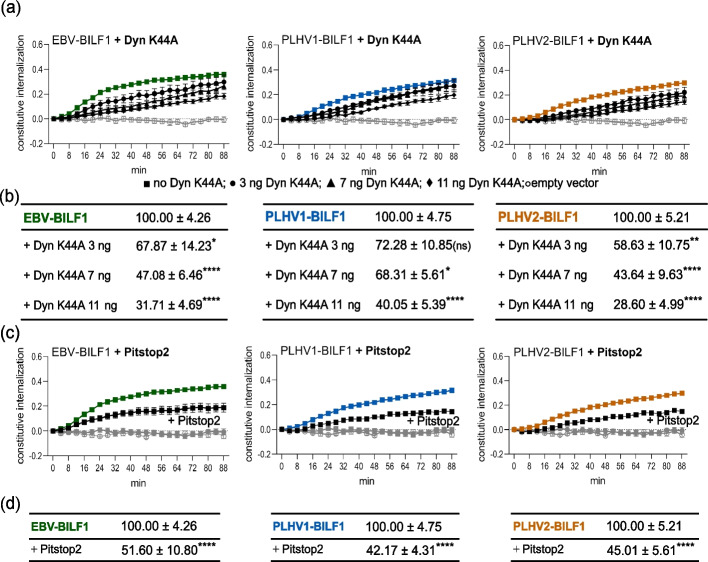
Internalization of BILF1 receptors depends on dynamin-1 and clathrin. SNAP-tagged EBV-BILF1 (green), SNAP-tagged PLHV1-BILF1 (blue), SNAP-tagged PLHV2-BILF1 (orange), and empty vector (gray) were expressed in HEK-293A cells and were either **a** co-transfected with different concentrations of Dyn K44A DNM (black circle 3 ng; black up-pointing triangle 7 ng; black diamond 11 ng) or **c** incubated with 5 µM Pitstop2 inhibitor (black circle or white circle for empty vector) or internalization buffer as a control (black square). Labeling with donor SnapLumi4-Tb was performed at 4 °C to prevent internalization prior to measurement. Internalization was measured every 4 min for 88 min at 37 °C. The graph curves represent the ratio between donor and acceptor. **b**, **d** The area under the curve (AUC) was calculated for each graph and was normalized to the AUC of each BILF1 receptor without addition of the Dyn K44A or Pitstop2 (100%) and empty vector (0%). Statistical differences were determined using Dunnett’s multiple comparisons two-way ANOVA test. *P < 0.05, **P < 0.01, *** P < 0.001, ****P < 0.0001

BILF1 receptors were transfected alone or together with increasing concentrations of Dyn K44A (Fig. 2). DNM Dyn K44A inhibited BILF1 internalization in a dose-dependent manner (Fig. 2a). For each internalization curve, the AUC parameter was calculated and was expressed relative to the AUC for each receptor in the absence of Dyn K44A (Fig. 2b). The effect of Dyn K44A on EBV-BILF1 and PLHV2-BILF1 was significant at all Dyn K44A concentrations showing 33‒42% (3 ng Dyn K44A), 53‒57% (7 ng Dyn K44A), and 69‒72% (11 ng Dyn K44A) lower internalization. PLHV1-BILF1 on the other hand, was significantly affected at the highest concentrations of Dyn K44A (7 and 11 ng per well), as the AUC values dropped to 68% and 40%, respectively. These results were supported by the surface expression of BILF1 receptors, which increased in correlation with the Dyn K44A concentration (Additional file 3a). Furthermore, we included the chemical inhibitor Pitstop2, to interfere with the formation of the clathrin coat at the membrane of the HEK-293A cells (Fig. 2c, d). Comparing the AUC of the BILF1 receptors in the absence and presence of Pitstop2, we observed comparable 49‒55% effects for the inhibitor on EBV-BILF1- and PLHV1-2 BILF1-mediated internalization (Fig. 2d), confirming the importance of intact clathrin-mediated endocytosis for BILF1 internalization.

### BILF1 internalization utilizes clathrin-coated pits, but is not dependent on β-arrestin recruitment

**Fig. 3:**
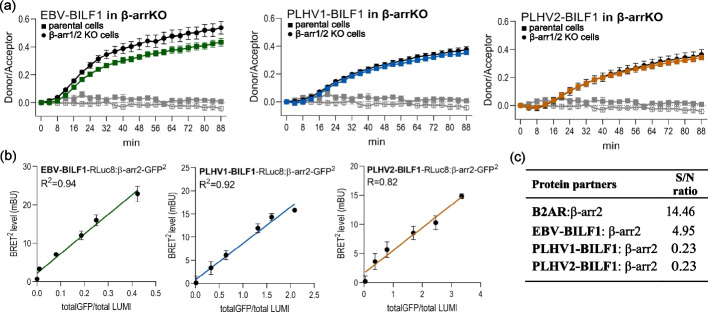
β-Arrestin recruitment is dispensable for the BILF1 receptor internalization. **a** SNAP-tagged EBV-BILF1 (green), SNAP-PLHV1-BILF1 (blue), SNAP-PLHV2-BILF1 (orange), and empty vector (gray) were expressed in parental HEK-293A cells (black square) and β-arrestin 1/2 knockout HEK-293A cells (Δβ-arr KO; black circle or white circle for empty vector). The curves represent the ratios between the donors and acceptors. Internalization was measured every 4 min for 88 min at 37 °C. **b** BILF1 receptors C-terminally tagged with RLuc8 were expressed in HEK-293A cells together with various concentrations of β-arr 2-GFP^2^. BRET2 values are plotted as a function of the ratio between the GFP^2^ (total fluorescence) and RLuc8 (total luminescence) signal. Results are presented as the mean (± SEM) from at least three independent experiments and are fitted using a simple linear regression equation (*R*). **c** The table presents the results from the informational spectrum method (ISM), determining the interaction of BILF1 receptors or the β2 adrenergic receptor (β2-AR) with β-arrestin (S/N ratio). A lower S/N ratio indicates lower interaction affinity

Previous studies on GPCRs showed that β-arrestins are not necessarily required for clathrin-mediated endocytosis [52, 53]. Therefore, we aimed to further investigate the involvement of β-arrestins in BILF1 internalization (Fig. 3). First, we compared the internalization of BILF1 receptors in HEK-293A (parental) cells and CRISPR/Cas9-modified β-arrestin 1/2 knockout cells (Δβ-arr1/2 KO) (Fig. 3a). The absence of β-arrestins in the Δβ-arr 1/2 KO cells did not affect receptor-mediated internalization by the BILF1 receptors, supporting the idea that, for BILF1 receptor internalization, β-arrestins are not required. As a control, we included the GIP-R activated by hormone GIP (Additional file 4a), which has been previously shown to require β-arrestin for its internalization [54]. To further understand the interaction of BILF1 receptors with β-arrestin, we performed BRET2 experiments (Fig. 3b) to determine the potential interaction of GFP^2^-βarr2 with RLuc8-tagged BILF1 receptors. Similarly, BRET2 saturation experiments have been previously performed, confirming the interaction between β-arrestin 2 and human GLP-1R [55]. Here, increasing concentrations of GFP^2^-βarr2 co-transfected with constant concentrations of RLuc8-BILF1 receptors, induced the so-called bystander BRET, presented as a simple linear regression curve (*R*^2^ > 0.8). The result was comparable to the finding of the control experiment, wherein we measured the interactions between BILF1 receptors and the 17aa membrane insert (*R*^2^ > 0.9) (Additional file 4b). These results showed random, nonspecific interactions between the investigated molecules.

Experimental evidence was supported by the ISM, a virtual spectroscopy method, to investigate protein–protein interactions and analyze protein structure–function relationships. A common peak corresponding to IS frequency *F*(0.216) (Additional file 1) determined here represents physicochemical characteristics based on the structural motif distribution of the GPCR and vGPCRs and corresponds to the biological function of the protein. To estimate the interaction affinity between BILF1 receptors and β-arrestin 2, we calculated the signal-to-noise ratio (S/N) (Fig. 3c) at the characteristic frequency for the interaction with β-arrestin 2 [39]. A lower S/N ratio suggests a lower interaction affinity between tested protein partners, as shown previously [35, 39]. The calculated ratios for the interactions with β-arrestin 2 are lower for BILF1 receptors (Fig. 3c) than for the interactions with the cellular β2 adrenergic receptor (β2-AR), a GPCR known for its β-arrestin interaction [56], suggesting a lower interaction affinity of the BILF1 receptors for β-arrestin 2. These results all support the observations that showed that BILF1 receptors internalize independently of β-arrestin. Importantly, the characteristic frequency *F*(0.216) showed that there was high interaction affinity (high S/N ratio) between all BILF1 receptors and AP-2. Furthermore, results of the immunoprecipitation assay confirmed this interaction for BILF1 receptors (Additional file 5), supporting the involvement of the clathrin-mediated pathway in BILF1 receptor endocytosis (Table 1).

**Table 1:** Interaction (S/N ratio) of BILF1 receptors with AP-2 determined using the informational spectrum method (ISM)

Protein partners	S/N ratio at *F*(0.216)^a^
β2-AR:AP-2	6.48
EBV-BILF1:AP-2	9.42
PLHV1-BILF1:AP-2	12.42
PLHV2-BILF1:AP-2	13.12

^a^ A lower S/N ratio indicates lower interaction affinity

### Caveolin-1 contributes to BILF1-mediated internalization

**Fig. 4:**
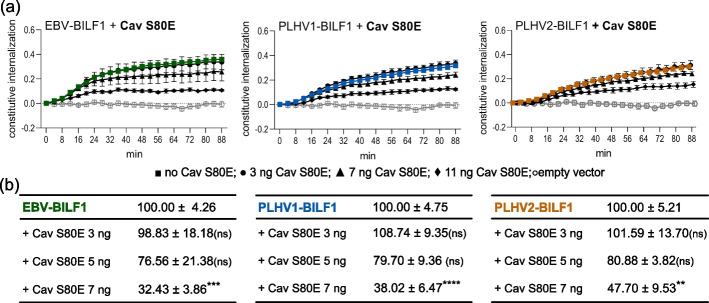
**Internalization of BILF1 receptors depends on caveolin-1 function.** **a** SNAP-EBV-BILF1 (green), SNAP-PLHV1-BILF1 (blue), SNAP-PLHV2-BILF1 (orange), and empty vector (gray) were expressed in HEK-293A cells together with different concentrations of Cav S80E DNM (black circle 3 ng; black up-pointing triangle 5 ng; black diamond 7 ng). Labeling with the donor SnapLumi4-Tb was performed at 4 °C to prevent any internalization prior to the measurement. Internalization was measured every 4 min for 88 min at 37 °C. The graph curves represent the ratios between donors and acceptors. **b** The area under the curve (AUC) was calculated for each curve and was normalized to each BILF1 receptor’s AUC in the absence of Cav S80E (100%) and empty vector (0%). Statistical differences were determined by Dunnett’s multiple comparison two-way ANOVA test. **P < 0.01, *** P < 0.001, ****P < 0.0001

Previous studies reported alternative pathways for vGPCR and GPCR endocytosis. To investigate the involvement of caveolin-1 in BILF1 receptor internalization, we used a DNM of caveolin-1 (Cav S80E) [34], which was previously found to be retained in the endoplasmic reticulum in a complex with caveolin-2 and to disrupt the caveolae formation at the membrane [57]. Again, we performed the FRET-based RT internalization assay, with SNAP-tagged BILF1 receptors in the absence or presence of increasing concentrations of Cav S80E (Fig. 4a).

The largest effect of the Cav S80E was observed with EBV-BILF1- and PLHV1-BILF1-mediated endocytosis, where the addition of 7 ng per well of the mutant resulted in 62‒68% lower AUC values. For PLHV2 BILF1, the co-transfection resulted in a ~ 53% lower AUC (Fig. 4b). It is important to note that the addition of 7 ng per well Cav S80E resulted in a decrease in BILF1 receptor expression (Additional file 3b). Furthermore, using the ISM, we showed that there was a high S/N ratio and thereby high interaction affinity between all BILF1 receptors and caveolin-1, confirming the involvement of this protein in BILF1 receptor trafficking (Table 2).

**Table 2:** Interaction (S/N ratio) of BILF1 receptors with caveolin-1 determined using the informational spectrum method (ISM)

Protein partners	S/N ratio at *F*(0.357)^a^
**EGF-R:**caveolin-1	15.29^b^
**EBV-BILF1:**caveolin-1	17.46
**PLHV1-BILF1:**caveolin-1	12.86
**PLHV2-BILF1:**caveolin-1	15.61

^a^A lower S/N ratio indicates lower interaction affinity

^b^Interaction of caveolin-1 and epidermal growth factor receptor (EGF-R) was used as a positive control [58]

### BILF1 receptors interact with the Rab7 protein, a marker for lysosomes

**Fig. 5:**
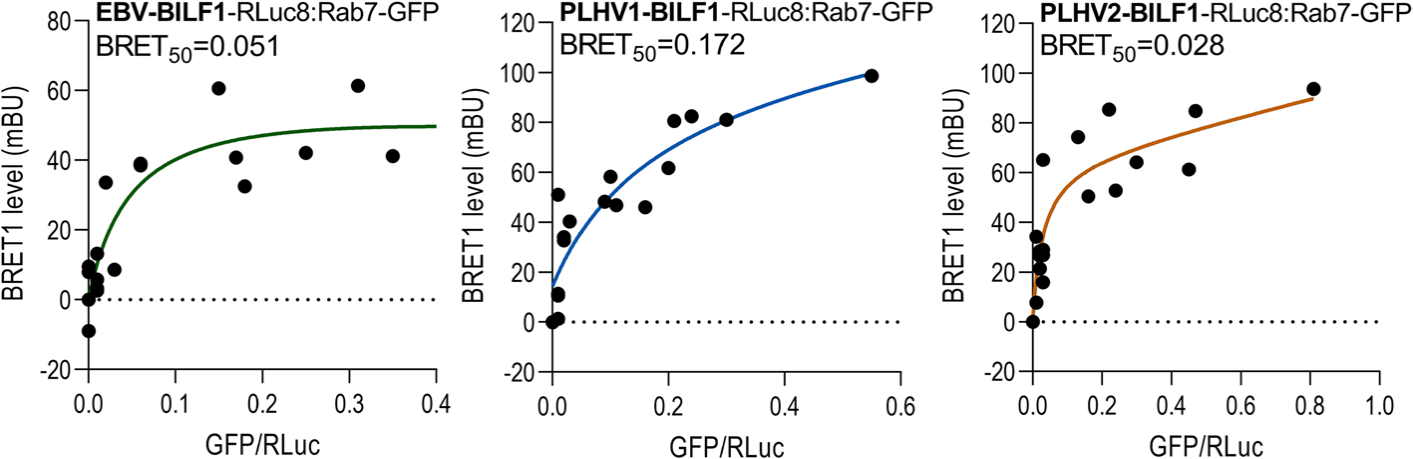
BILF1 receptors interact with Rab7 protein. BILF1 receptors C-terminally tagged with RLuc8 and various concentrations of Rab7-EGFP protein were expressed in HEK-293A cells. BRET1 values are plotted as a function of the ratio between the GFP (total fluorescence) and RLuc8 (total luminescence) signal. Results are fitted using a nonlinear regression equation. BRET_50_ represents the acceptor/donor ratio at 50% of the BRET_max_, indicating the relative affinity of the acceptor (BILF1-RLuc8) for the donor molecules (Rab7-EGFP). Graphs represent the mean (± SEM) from at least three independent experiments

After confirming their constitutive internalization from the plasma membrane and subsequently demonstrating that there was an intracellular pool of receptors, which indicates their ability to recycle (Fig. 1), we further investigated whether these receptors follow the degradation pathway in lysosomes. BRET1 studies were conducted to investigate the possible interactions between RLuc8-tagged BILF1 receptors and the EGFP-tagged Rab7 protein, a marker of late endosomes/lysosomes [59, 60]. To follow the intracellular trafficking of BILF1 receptors after constitutive internalization, a BRET1 saturation assay was used, and the interactions observed between a constant BILF1 receptor concentration and an increasing Rab7 concentration, as previously described [61]. Saturation was reached for all BILF1 receptors in the presence of increasing amounts of Rab7, resulting in hyperbolic saturation curves with BRET_50_ values of 0.028‒0.172 (Fig. 5). The result indicates the constitutive interaction of BILF1 receptors with late endosomes/lysosomes and therefore suggests that at least a fraction of these receptors are degraded after their internalization from the cell surface.

## Discussion

In this investigation we have confirmed and further described the constitutive endocytosis of three BILF1 receptors encoded by the human EBV and porcine PLHV1 and 2. Using a novel real-time FRET-based internalization assay, we have demonstrated that the BILF1 receptors require intact clathrin-coat formation and dynamin-1 to function but do not require β-arrestin recruitment for their internalization, suggesting the involvement of a clathrin-mediated, β-arrestin-independent endocytic pathway. Further, the interaction of BILF1 with caveolin-1 suggests the involvement of this protein in BILF1 trafficking.

Constitutive internalization occurs when a membrane receptor transports to the cytoplasm independently of an extracellular ligand. This has been described for several vGPCRs [8, 15, 35, 62, 63] and endogenous GPCRs [53, 62]. Although constitutive internalization is a common feature in vGPCRs, its functional role remains poorly understood. However, different functions have been proposed, such as ensuring sufficient receptor expression at the membrane [63], representing basal constitutive receptor activity [64], involvement in ligand scavenging [65], and allowing intracellular endosomal signaling [66]. Besides, constitutive internalization has also been studied for the purposes of drug targeting, as in the case of HCMV-US28, wherein the receptor was targeted by a fusion toxin protein (FTP) to deliver the toxin to the infected cells and hence induce cell death [67–69].

Here, we have confirmed the constitutive internalization of EBV-BILF1 and PLHV1-2 BILF1 orthologs using a novel real-time FRET-based internalization assay, which allowed us to study the endocytic properties of these receptors in live HEK-293A cells. Low levels of receptor expression and low internalization ratios [15] observed for PLHV3-BILF1 made further interpretation of the endocytic properties difficult—and consequently, this receptor was not included in this study.

Internalization kinetics have been previously reported for other vGPCRs and GPCRs, and examples include thyrotropin-releasing hormone receptor (TRH) with rapid internalization (*t*_1/2_ of 2.2 min) [49], gonadotropin-releasing hormone (GnRH) receptor with slower agonist-induced internalization (*t*_1/2_ of 20 min) [49], and CXCR4 receptor with slow constitutive and rapid CXCL12-induced internalization (*t*_1/2_ values of ~ 15 min and ~ 5 min, respectively) [70]. Moreover, constitutive internalization of HCMV-US28 was comparable to the fast ligand-induced CXCR4 internalization [71]. Compared with the previously reported data, our results show slow constitutive internalization for EBV-BILF1 with a *t*_1/2_ of 20 min and even slower constitutive internalization for PLHV1-2-BILF1 with a *t*_1/2_ of 37‒45 min. A role of constitutive internalization for EBV-BILF1 has been proposed previously, suggesting that the receptor forms a complex with MHC-I molecules at the plasma membrane and induces its internalization, resulting in hindered immunorecognition by CD8^+^ T cells [12]. The kinetics of the BILF1 constitutive internalization reported here correspond to the ability of these receptors to downregulate MHC-I molecules [15], where EBV-BILF1 showed the highest impact on MHC-I expression in HEK-293 cells. However, to assign the biological function of constitutive internalization to the BILFs, further studies will be required.

Previous studies reported the use of Dyn K44A [34, 50, 72] and Pitstop2 [51, 73, 74] as means by which to investigate the clathrin-mediated pathway. In addition to the significant changes in BILF1 internalization induced by the inhibitors, we also observed an increase in receptor expression after the addition of Dyn K44A. The requirement for β-arrestin in the internalization process depends on the receptor and receptor’s mode of internalization [75]. Constitutive but not ligand-induced internalization of HCMV-US28 uses a β-arrestin-independent pathway, as was determined by the use of β-arrestin KO embryonic fibroblasts [52]. Furthermore, GPCR ADGRA3 (GPR125) internalizes constitutively through a clathrin-mediated β-arrestin-independent pathway [53]. Here, we report this finding to be characteristic of BILF1 internalization. Our data on internalization in β-arr KO cells were supplemented with the analysis of the protein interaction. The β-arrestin recruitment assay has been widely used and showed specific interactions of β-arrestin2 with the GLP-1 receptor [55], β2-AR [56], neurokinin-1 receptor (NK-1) [34], and D2 dopamine receptor [38]. We have observed only a nonspecific bystander BRET2, showing random interaction of BILF1 with β-arrestin 2. Based on the amino acid sequences of the receptors, the bioinformatics approach using ISM has suggested that there is no interaction with β-arrestin, which further confirms the exclusion of β-arrestin in BILF1-mediated internalization (and signaling). A direct interaction of AP2 with three motif types on GPCRs has been reported previously and was suggested as a mechanism that facilitates clathrin-mediated endocytosis in an arrestin-independent manner [19, 76]. The tyrosine (YXXΦ) receptor motif [77], the [DE]XXXL[LI] receptor motif [78, 79], and the polyarginine receptor motif [19] are all located in the cytoplasmic end of the GPCRs and interact with the µ2 adaptin subunit of AP2. In our previous publication [80], we have shown the existing tyrosine motifs on EBV-, PLHV1-, and PLHV2-BILF1 receptors using the eukaryotic linear motif (ELM) resource. Furthermore, the bioinformatics analysis in this study showed high interaction affinity between AP2 and BILF1 receptors, which supports the pathway proposed above.

The caveolae-mediated pathway has been described as an alternative pathway used by several GPCRs, viruses, and bacteria to enter the cell [81]. Despite their endocytic role, novel studies have reported new important, tissue-specific roles for caveolae including mechanoprotective roles [82–84], transport of fatty acids [85, 86], regeneration, mechanosensation [87], and a recently discovered role responding to oxidative stress and UV exposure [88, 89]. HEK-293 cells are known to express low levels of caveolin-1 and high levels of caveolin-2; however, the formation of flask-shaped caveolae has not yet been reported in this cell line. Here, a significant decrease in receptor internalization was observed in the presence of DNM Cav S80E, despite low caveolin-1 expression and lack of caveolae in HEK-293 cells. A similar observation has been reported previously by Lajoie et al. [90], as they reported the dynamin-dependent, raft-mediated endocytosis of the cholera toxin B subunit in Mgat5^−/−^ cells that express few or no caveolae, suggesting that caveolin-1 regulation of endocytosis does not depend on the formation of caveolae. In the same cell line, they also reported caveolin-1-mediated regulation of EGF-R diffusion and signaling [91]. In our study we have similarly shown the requirement for dynamin-1 and caveolin-1 in BILF1 internalization, albeit the level of caveolin-1 expression was found to be below the threshold for caveolae formation. The data from the bioinformatics approach also confirm a possible interaction between caveolin-1 and BILF1, with interaction affinity (S/N ratio) comparable to caveolin-1:EGF-R. This indicates a potential pathway for BILF1 internalization; however, the precise function of this interaction is not yet understood and will require further investigation.

Furthermore, increasing concentrations of Cav S80E reduced BILF1 surface expression. This suggested that the receptor is retained intracellularly after impairment of the caveolin function. Previously, a chaperone function of caveolin was proposed and was shown to be important for several GPCRs. Using the DNM of caveolin-1 and generation of receptor mutants with modified caveolin binding sites, the impaired surface expression was shown for glucagon-like peptide 1 (GLP-1) receptor, insulin (IR) receptor, excitatory amino acid carrier 1 (EAAC1), and type 1 receptor for angiotensin II (AT1), suggesting that these receptors require functional caveolin-1 to be expressed at the cell surface [24, 92–95].

After internalization from the plasma membrane, GPCR trafficking leads to a recycling or degradation pathway. Previous studies on KSHV-ORF74 showed trafficking of the vGPCR through both recycling and late endosomes/lysosomes [60]. Here, we have also observed both pathways to be involved in BILF1 intracellular trafficking. A previous study by Zuo et al. has proposed that BILF1 forms a complex with MHC-I molecules at the plasma membrane and that this induces complex internalization [12]. They also showed that treatment with a lysosomal inhibitor could reveal a marked pool of intracellularly located MHC-I molecules in BILF1-transfected cells [12]. Our results indicated the involvement of the degradation pathway for EBV-BILF1, and therefore support these previous findings. Moreover, the presumed ability of BILF1 receptors to recycle back to the plasma membrane may serve to ensure that there is adequate BILF1 expression for MHC-I downregulation. However, a more detailed investigation into the functional consequence of BILF1 internalization and trafficking is required.

## Conclusions

In summary, we have shown that BILF1 receptors exhibit slow constitutive internalization through a β-arrestin-independent, clathrin-mediated pathway. Furthermore, we have also identified that caveolin-1 may potentially be involved in BILF1 trafficking. After internalization, the BILF1 receptors are presumably processed through both recycling and degradation pathways. The results can serve as a foundation for future studies to explore a PLHV-infected porcine model as a mechanism to study BILF1 as a potential therapeutic target in EBV-related disease.

### Supplementary Information

**Additional file 1.** Informational spectrum (IS) frequency representing common informational characteristics determined using the informational spectrum method (ISM). Common peak corresponding to IS frequency *F*(0.216) represents physicochemical characteristics based on the structural motif distribution of the BILF1 receptors and corresponds to the biological function of the protein.

**Additional file 2.** Principle of the real-time FRET-based method and the intracellular receptor pool calculation. Schematic representation of the principle used to calculate the intracellular receptor pool using RT-FRET-based internalization method.

**Additional file 3.** BILF1 receptor expression in HEK-293 cells co-transfected with caveolin and dynamin DNMs and in β-arrestin 1/2 KO cells. The figure shows the expression of BILF1 receptors in HEK-293A or βarr1/2 KO cells, which was measured in parallel with internalization using real-time FRET-based method. The presented expression was measured at timepoint 0 min for all BILF1 receptors in different conditions.

**Additional file 4.** Control experiments for β-arrestin-mediated internalization and β-arrestin recruitment. Figure shows the results from real-time FRET-based internalization assay for control GIP-R and the control BRET2 saturation assay, where we co-expressed BILF1 receptors together with a membrane insert in HEK-293 cells. The linear regression curve represents random collision between surface-expressed BILF1 receptors and membrane insert.

**Additional file 5.** Immunoprecipitation experiments. Immunoprecipitation experiments, confirming the interaction between BILF1 receptors and AP-2.

## Data Availability

All data generated or analyzed during this study are included in this published article and its additional information files.

## Abbreviations

vGPCR: Viral G-protein-coupled receptor
EBV: Epstein–Barr virus
PLHV1-2: Porcine lymphotropic herpesvirus 1 and 2
MHC-I: Major histocompatibility complex I
AP-2: Adaptor protein 2
SV40: Simian virus 40
HEK-293: Human embryonic kidney cells 293
KO: Knockout
ISM: Informational spectrum method
BRET1: Bioluminescence resonance energy transfer
AUC: Area under the curve
FRET: Fluorescence resonance energy transfer
RT: Real time
*t*_1/2_: Half-time
S/N: Signal-to-noise ratio
β2-AR: β2-Adrenergic receptor
DNM: Dominant-negative mutant
KSHV: Kaposi’s sarcoma herpesvirus
HCMV: Human cytomegalovirus
FTP: Fusion toxin protein
TRH: Thyrotropin-releasing hormone receptor
GnRH: Gonadotropin-releasing hormone
ELM: Eukaryotic linear motif resource
GLP-1: Glucagon-like peptide 1
IR: Insulin receptor
EAAC1: Excitatory amino acid carrier 1
AT1: Type 1 receptor for angiotensin II
