# Correction: Patterns of human and porcine gammaherpesvirus-encoded BILF1 receptor endocytosis

**DOI:** 10.1186/s11658-023-00512-2

**Published:** 2023-11-24

**Authors:** Maša Mavri, Sanja Glišić, Milan Senćanski, Milka Vrecl, Mette M. Rosenkilde, Katja Spiess, Valentina Kubale

**Affiliations:** 1 Institute for Preclinical Sciences, Veterinary Faculty, Ljubljana, Slovenia; 2 Center for Multidisciplinary Research, Institute of Nuclear Sciences VINCA, University of Belgrade, Belgrade, Serbia; 3 Department of Biomedical Sciences, Faculty of Health and Medical Sciences, University of Copenhagen, Copenhagen, Denmark; 4 Present Address: Department of Virus and Microbiological Special Diagnostics, Statens Serum Institute, Copenhagen, Denmark

**Correction : Cellular & Molecular Biology Letters (2023) 28:14** **https://doi.org/10.1186/s11658-023-00427-y**

Following publication of the original article [1], the authors updated the Funding section.

The original article has been corrected.
