# Supplementary material for: Patterns of human and porcine gammaherpesvirus-encoded BILF1 receptor endocytosis
Source: Cell Mol Biol Lett. 2023 Feb 21;28:14. doi: 10.1186/s11658-023-00427-y (PMC9942385; doi:10.1186/s11658-023-00427-y)
Supplement: Supplementary file 2 — Additional file 2. Principle of the real-time FRET-based method and the intracellular receptor pool calculation. Schematic representation of the principle used to calculate the intracellular receptor pool using RT-FRET-based internalization method. [file 11658_2023_427_MOESM2_ESM.pdf]

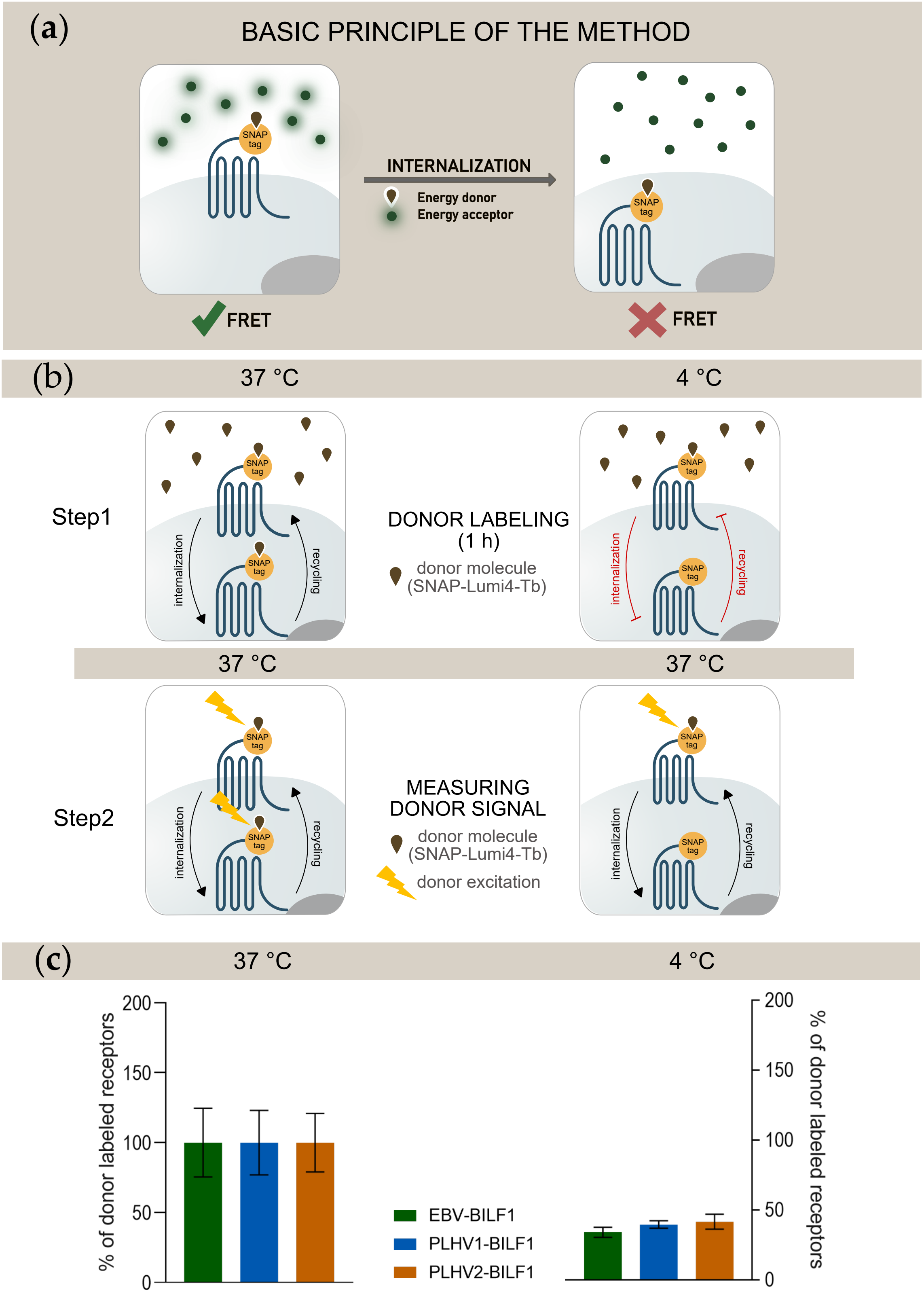

Additional file 2. Principle of the RT-FRET based method and the intracellular receptor pool calculation. (**a**) SNAP-tagged receptor constructs are first labeled with a donor molecule for 1 hour. The unbound donor is then washed, and the acceptor molecule is added to the cells. The internalization measurement is performed every minute for 90 minutes at 37 °C. (**b**) Donor labeling was performed at both 37 °C (allowing normal internalization and recycling) and at 4 °C (preventing internalization and recycling). The labeling at 4 °C thus resulted in the labeling of surface expressed receptors only, whereas labeling at 37 °C allowed the labeling of all receptors cycling from the plasma membrane to the cell interior. (**c**) The graph represents the differences in donor signal between the two conditions. The difference accounts for the estimated number of receptors trafficked to the plasma membrane.
