# Supplementary material for: Patterns of human and porcine gammaherpesvirus-encoded BILF1 receptor endocytosis
Source: Cell Mol Biol Lett. 2023 Feb 21;28:14. doi: 10.1186/s11658-023-00427-y (PMC9942385; doi:10.1186/s11658-023-00427-y)
Supplement: Supplementary file 3 — Additional file 3. BILF1 receptor expression in HEK-293 cells co-transfected with caveolin and dynamin DNMs and in β-arrestin 1/2 KO cells. The figure shows the expression of BILF1 receptors in HEK-293A or βarr1/2 KO cells, which was measured in parallel with internalization using real-time FRET-based method. The presented expression was measured at timepoint 0 min for all BILF1 receptors in different conditions. [file 11658_2023_427_MOESM3_ESM.pdf]

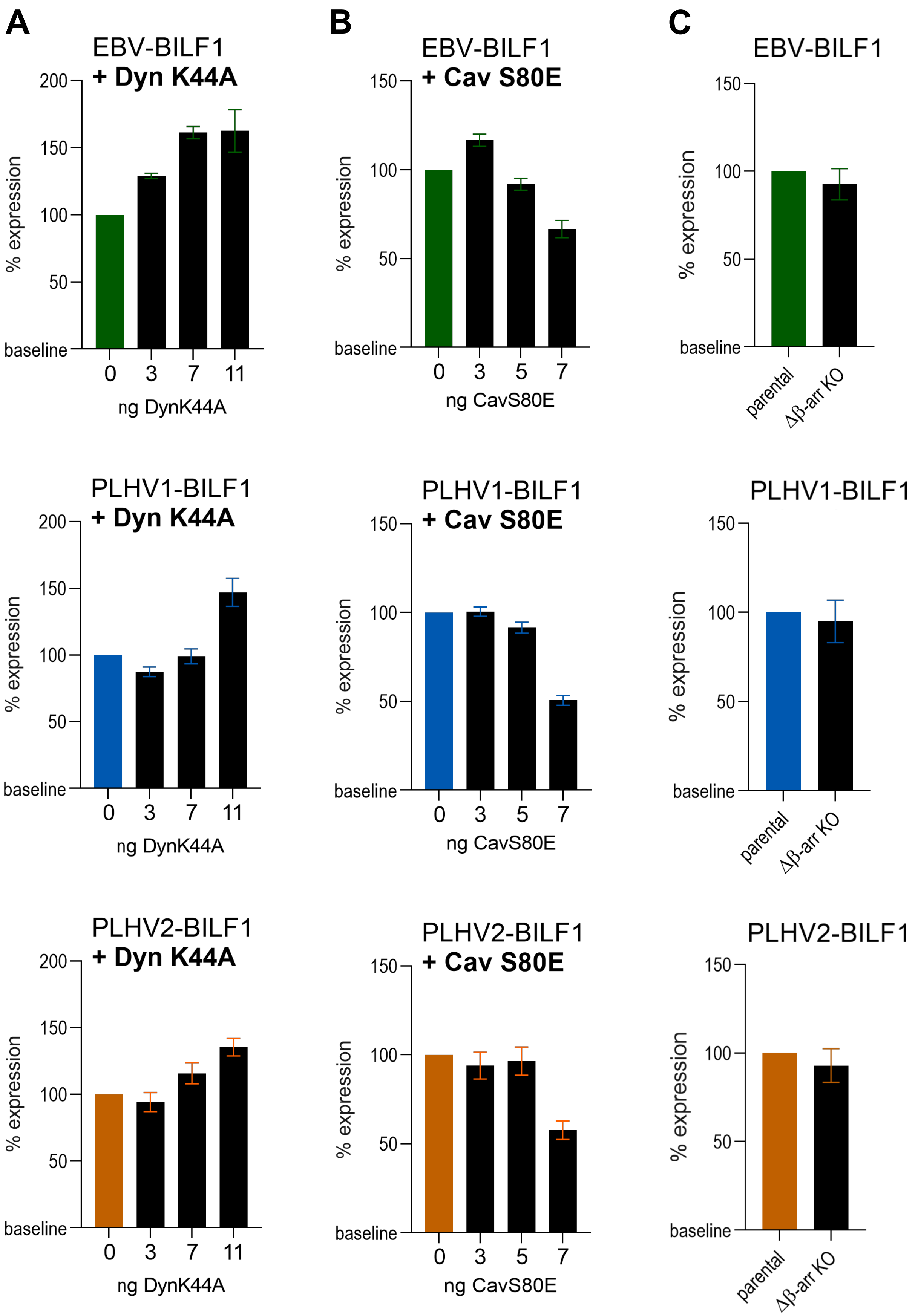

Additional file 3. BILF1 receptor expression in HEK-293 cells co-transfected with caveolin and dynamin DNMs and in β-arrestin 1/2 KO cells. A and B) SNAP-EBV-BILF1 (green), SNAP-PLHV1-BILF1 (blue) and SNAP-PLHV2-BILF1 (orange) were expressed in HEK-293A cells and were co-transfected with different concentrations of A) Dyn K44A DNM or B) Cav S80E DNM C) BILF1 orthologues were transfected in HEK-293A parental and β-arr 1/2 KO cells. Donor values at time point 0 were used to determine and compare receptor expression. Data are shown as the mean ± SEM from at least three independent experiments carried out in triplicate.
