# Supplementary material for: Patterns of human and porcine gammaherpesvirus-encoded BILF1 receptor endocytosis
Source: Cell Mol Biol Lett. 2023 Feb 21;28:14. doi: 10.1186/s11658-023-00427-y (PMC9942385; doi:10.1186/s11658-023-00427-y)
Supplement: Supplementary file 4 — Additional file 4. Control experiments for β-arrestin-mediated internalization and β-arrestin recruitment. Figure shows the results from real-time FRET-based internalization assay for control GIP-R and the control BRET2 saturation assay, where we co-expressed BILF1 receptors together with a membrane insert in HEK-293 cells. The linear regression curve represents random collision between surface-expressed BILF1 receptors and membrane insert. [file 11658_2023_427_MOESM4_ESM.pdf]

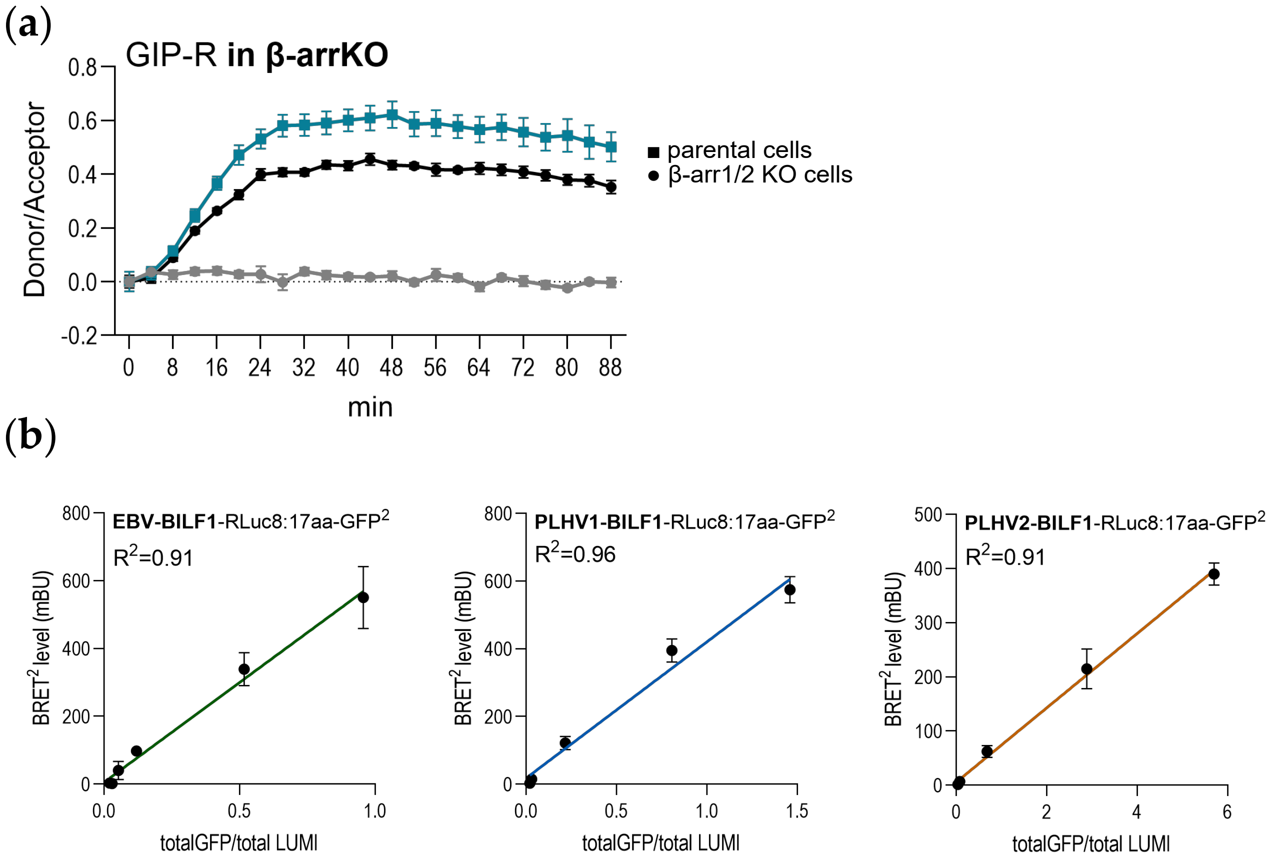

Additional file 4. Control experiments for β-arrestin mediated internalization and β-arrestin recruitment. a) Graph shows the internalization of GIP-R in presence of its agonist GIP. GIP-R is known for its β-arrestin mediated internalization. The graph curves represent the ratio between donor and acceptor. b) Graphs show random collisions between RLuc8-tagged BILF1 orthologues and various concentrations of membrane inserted GFP217aa constructs in HEK-293 cells. BRET2 values are plotted as a function of the ratio between the GFP2 (total fluorescence) and RLuc8 (total luminescence) signal. Results are presented as the mean (± SEM) from at least three independent experiments and are fitted using a simple linear regression equation.
