# Supplementary material for: Patterns of human and porcine gammaherpesvirus-encoded BILF1 receptor endocytosis
Source: Cell Mol Biol Lett. 2023 Feb 21;28:14. doi: 10.1186/s11658-023-00427-y (PMC9942385; doi:10.1186/s11658-023-00427-y)
Supplement: Supplementary file 5 — Additional file 5. Immunoprecipitation experiments. Immunoprecipitation experiments, confirming the interaction between BILF1 receptors and AP-2. [file 11658_2023_427_MOESM5_ESM.pdf]

(a)

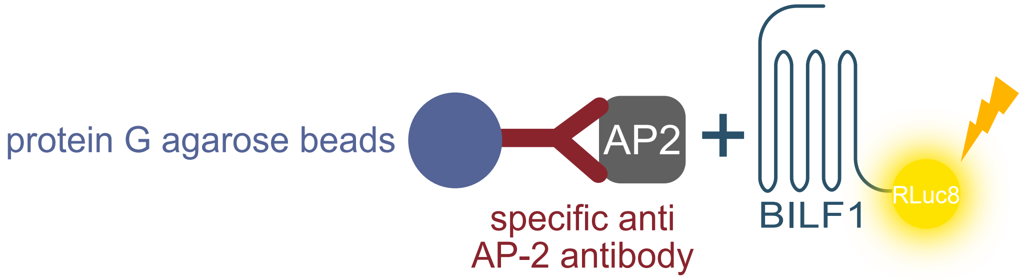

(b)

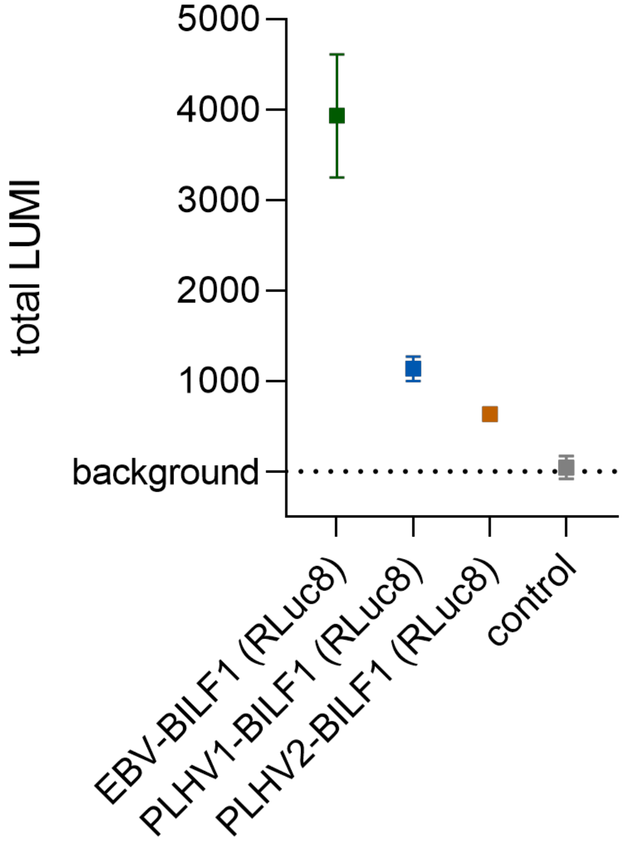

Additional file 5. Immunoprecipitation experiments, confirming the interaction between BILF1 receptors and AP-2. a) A schematic representation of the immunoprecipitation method used to measure the interaction between AP-2 and BILF1 receptor. b) Immunoprecipitation experiments were performed in HEK-293 cells transfected with RLuc8-tagged BILF1 receptors. Specific antibodies agains endogenous AP-2 and additional protein G agarose beads were used to separate the BILF1/AP2 complex. RLuc8 signal (total luminescence) was measured to detect the BILF1 receptor in the isolated fraction.
