# Supplementary material for: Patterns of human and porcine gammaherpesvirus-encoded BILF1 receptor endocytosis
Source: Cell Mol Biol Lett. 2023 Feb 21;28:14. doi: 10.1186/s11658-023-00427-y (PMC9942385; doi:10.1186/s11658-023-00427-y)
Supplement: Supplementary file 1 — Additional file 1. Informational spectrum (IS) frequency representing common informational characteristics determined using the informational spectrum method (ISM). Common peak corresponding to IS frequency F(0.216) represents physicochemical characteristics based on the structural motif distribution of the BILF1 receptors and corresponds to the biological function of the protein. [file 11658_2023_427_MOESM1_ESM.pdf]

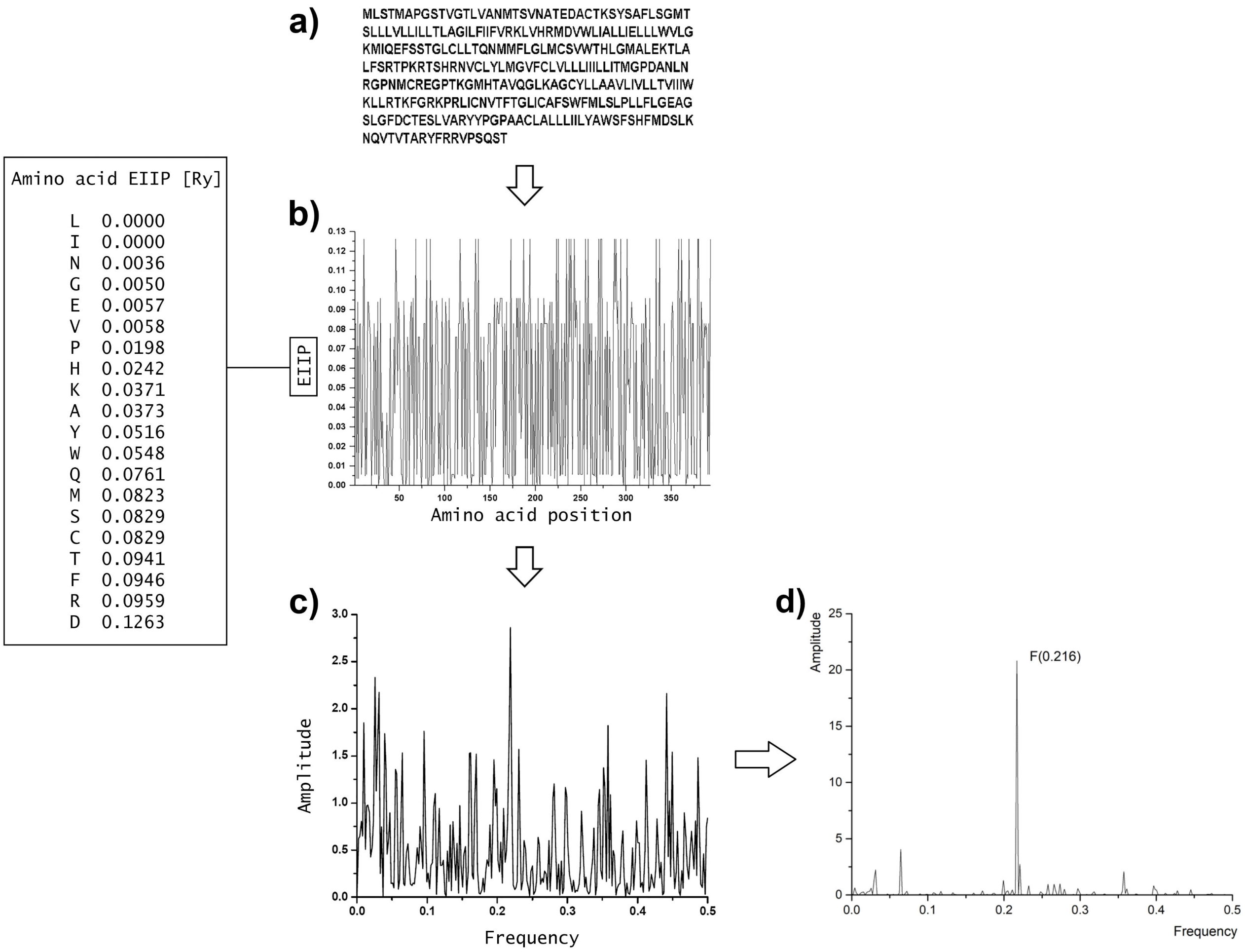

Additional file 1. Basic principle of Informational spectrum method (ISM). The ISM approach consists of two steps. a) The amino acids (single letter amino acid code) in the primary protein structure are represented by b) numbers corresponding to the electron-ion interaction potential (EIIP). c) The obtained numerical sequence is transformed by Fourier transformation into an informational spectrum (IS). Each peak in the IS represents particular information encoded in the protein's primary structure. Proteins that interact with a common target have been shown to share common frequencies in their IS. d) ISM was used to investigate important common informational characteristics of the BILF1 orthologues, and the common informational characteristic was found to be represented by the consensus IS frequency F(0.216) in consensus Informational spectrum (CIS).
